# Top-Down Lipidomics Reveals Ether Lipid Deficiency in Blood Plasma of Hypertensive Patients

**DOI:** 10.1371/journal.pone.0006261

**Published:** 2009-07-15

**Authors:** Juergen Graessler, Dominik Schwudke, Peter E. H. Schwarz, Ronny Herzog, Andrej Shevchenko, Stefan R. Bornstein

**Affiliations:** 1 Department of Internal Medicine III, Carl Gustav Carus Medical School, Technical University Dresden, Dresden, Germany; 2 Max Planck Institute of Molecular (MPI) of Molecular Cell Biology and Genetics, Dresden, Germany; University of Las Palmas de Gran Canaria, Spain

## Abstract

**Background:**

Dyslipoproteinemia, obesity and insulin resistance are integrative constituents of the metabolic syndrome and are major risk factors for hypertension. The objective of this study was to determine whether hypertension specifically affects the plasma lipidome independently and differently from the effects induced by obesity and insulin resistance.

**Methodology/Principal Findings:**

We screened the plasma lipidome of 19 men with hypertension and 51 normotensive male controls by top-down shotgun profiling on a LTQ Orbitrap hybrid mass spectrometer. The analysis encompassed 95 lipid species of 10 major lipid classes. Obesity resulted in generally higher lipid load in blood plasma, while the content of tri- and diacylglycerols increased dramatically. Insulin resistance, defined by HOMA-IR >3.5 and controlled for BMI, had little effect on the plasma lipidome. Importantly, we observed that in blood plasma of hypertensive individuals the overall content of ether lipids decreased. Ether phosphatidylcholines and ether phosphatidylethanolamines, that comprise arachidonic (20∶4) and docosapentaenoic (22∶5) fatty acid moieties, were specifically diminished. The content of free cholesterol also decreased, although conventional clinical lipid homeostasis indices remained unaffected.

**Conclusions/Significance:**

Top-down shotgun lipidomics demonstrated that hypertension is accompanied by specific reduction of the content of ether lipids and free cholesterol that occurred independently of lipidomic alterations induced by obesity and insulin resistance. These results may form the basis for novel preventive and dietary strategies alleviating the severity of hypertension.

## Introduction

Hypertension, a key component of the metabolic syndrome, is a major risk factor for cardiovascular disease and mortality [1]–[3]. Population-based studies indicate that the prevalence of hypertension is about 20% in normal weight individuals and more than 50% in obese individuals [4], [5]. Hypertension is frequently accompanied by metabolic disorders such as elevated levels of triglycerides and LDL cholesterol, lowered levels of HDL cholesterol, as well as insulin resistance [5]. Early hypertension is associated with increased epicardial and visceral fat deposits [6], [7], the cells which secrete adipokines modulating both mineralocorticoid secretion and sympathetic nervous activity, thus contributing to increased blood pressure. Dyslipidemia triggers endothelial dysfunction further contributing to the development of hypertension [8]. As lipid-lowering drugs contribute to blood pressure normalization, treatment with statins might prevent coronary and stroke events in hypertensive patients who have average or lower than average levels of blood plasma cholesterol [9], [10].

Traditionally, clinicians monitor lipid homeostasis in blood plasma *via* integral indices reflecting the cholesterol content (total, HDL-, LDL-) and total triglycerides, while phenotypic manifestations of obesity are estimated by the body mass index (BMI) and waist-to-hip ratio (WHR). While obesity strongly impacts all lipid homeostasis indices, it is unclear whether the plasma lipidome is altered specifically and independently of other components of the metabolic syndrome in patients with hypertension, and, if so, what lipid species or classes might be affected. Although previous observations indicated that such links might exist, its molecular basis remains unclear. We therefore hypothesized that systematic analysis of the blood plasma lipidome at the level of individual molecular species might help to elucidate such mechanism.

A palette of mass spectrometry-based technologies has been applied for lipidome profiling. Lipids can be pre-separated by liquid chromatography (LC) and identified through tandem mass spectrometry (LC-MS/MS) by their accurately determined masses and retention times [11]–[16]. Alternatively, shotgun lipidomics relies upon direct infusion of total lipid extract into a tandem mass spectrometer. Species of individual lipid classes are detected and quantified using precursor and neutral loss scans specific for their common structural elements [17]–[20]. Top-down shotgun strategy takes advantage of high mass resolution of modern mass spectrometers, such that lipids of major classes could be recognized by accurately determined precursor masses with no recourse to MS/MS. Although individual molecular species are not identified directly, as by complementary bottom-up lipidomics [21]–[23], top-down lipidomics has demonstrated potential in high-throughput screens [24].

Lipidomics screening of plasma in a small cohort of monozygotic twins discordant for obesity revealed a significant increase in lysophosphatidylcholines (LPC) and a decrease in ether phospholipids in obese individuals [25]. Predictably, gradual weight loss decreased the overall level of serum triacylglycerols (TAG) [26]. The improvement of insulin sensitivity during diet-induced weight loss was also accompanied by diminishing the levels of phosphatidylcholines (PC) and phosphatidylethanolamines (PE), while the levels of LPC's and sphingomyelines (SM) remained unchanged [26]. Whether hypertension is associated with characteristic plasma lipidomic changes has not yet been investigated.

A case-controlled study was designed to address the interrelationship between hypertension and blood plasma lipidome. Valid population sampling was achieved by the random recruitment of individuals from an ongoing prospective study and by the enforcement of strict exclusion criteria, such as on-going treatment with antihypertensive drugs, indications of inflammatory processes, liver and kidney diseases, as well as diabetes mellitus. The impact of variable genetic and hormonal background was addressed as follows: first, by engaging a larger subject population; second, by restricting the study to men; and third, by accounting for the lipidomic impact of known potent hypertension-related factors, such as obesity and insulin resistance and considering these factors as covariates in multivariate (MANOVA) and univariate (ANCOVA) models of analysis of variance.

The top-down lipidomics approach monitored the abundance of 95 lipid species originating from 10 lipid classes in the plasma of 70 male individuals, 19 of whom were hypertensive. We were able to demonstrate that hypertension was specifically associated with reduced levels of free plasma cholesterol and ether lipids, in particular with ether phosphatidylcholines (PC-O) and ether phosphatidylethanolamines (PE-O), while other lipid classes (including TAGs) remained practically unaffected.

## Materials and Methods

### Subjects

Male subjects were randomly selected from the ongoing PRAEDIAS prevention study in the Department of Internal Medicine III at Carl Gustav Carus Medical School, Technical University Dresden as previously described [27]. Each participant provided written informed consent and the study was approved by the Dresden Ethics Committee (EK139092001) in accordance with the Declaration of Helsinki principles. All test subjects underwent a standardised clinical examination including a comprehensive metabolic characterization. Only individuals who did not manifest acute inflammatory processes (C-reactive protein <5 mg/L), diabetes mellitus or severe renal or hepatic diseases, participated in the study. Subjects should not have received antihypertensive medication or therapy that might have affected lipid metabolism. The study population consisted of 70 men including 19 subjects with hypertension. Basal anthropometric and clinical data are presented in Supplemental Table S1 (Supplementary Materials).

Blood samples for lipid profiling were taken after overnight fasting and EDTA-plasma was prepared by 10 min centrifugation at 4°C and 3000 g. All samples were immediately shock-frozen in liquid nitrogen and stored at −80°C until analysed.

Homeostasis model assessment of insulin resistance (HOMA-IR) index was calculated as (fasting insulin [µU/mL]×fasting glucose [mM])/22.5 [28].

Blood pressure was measured in accordance with WHO guidelines. The diagnosis of hypertension was based on systolic blood pressure ≥140 mmHg and/or diastolic blood pressure 90≥mmHg.

### Clinical chemistry indices

Plasma triglycerides, total cholesterol, HDL and LDL cholesterol were determined by standard enzymatic methods on a MODULAR analyser (Roche, Indianapolis, IN), free fatty acid on a COBAS MIRA analyser (Global Medical Instrumentation Inc, Ramsey, MN), and plasma glucose on a DX80 analyser (Beckman-Coulter, Fullerton, CA). HbA1C was measured by HPLC (Bio-Rad Laboratories, Richmond, CA). Plasma insulin levels were determined by an enzyme-linked immunosorbent assay (Asbach Medical Products, Obrigheim, Germany).

### Chemicals and lipid standards

Synthetic lipid standards were purchased from Avanti Polar Lipids, Inc. (Alabaster, AL). Water (LC-MS grade) was purchased from Fisher Scientific (Loughborough, United Kingdom); chloroform, methanol and ammonium acetate were of Liquid Chromatography grade and purchased from Fluka (Buchs SG, Switzerland). Methyl-*tert*-butylether (MTBE) and 2-propanol were purchased from Sigma-Aldrich Chemie GmbH (Munich, Germany).

### Lipid extraction

Plasma samples were thawed and extracted with MTBE as described in [20]. Briefly, 20 µL of EDTA plasma was placed in a 2 mL vial (Eppendorf, Hamburg, Germany). 320 µL of the internal standards mixture consisting of 1,2-di-O-octadecyl-*sn*-glycero-3-phosphocholine, 1,2-di-O-phytanyl-*sn*-glycero-3-phosphoethanolamine and N-heptadecanoyl-D-*erythro*-sphingosylphosphorylcholine with a concentration of 3.2 nM for each lipid in 94% methanol was added and the solution was thoroughly mixed. Afterwards, 1 mL of MTBE was added and the mixture was vortexed at 20°C for one hour. Then 250 µL of water were added and tube thoroughly vortexed. After centrifuging for 1 minute at 4000 rpm on a Minispin centrifuge (Eppendorf, Hamburg, Germany,) 800 µL of the upper organic phase was transferred into a new vial and stored at −20°C until analysis. For mass spectrometric analysis, 8 µL of the extract were diluted with 80 µL CHCl_3_/MeOH/2-propanol 1/2/4 (v/v/v) containing 7.5 mM ammonium acetate in a well of a 96 well plate (Eppendorf, Hamburg, Germany) and then sealed with aluminum foil.

### Mass spectrometric analysis

Mass spectrometric analysis was performed on a hybrid LTQ Orbitrap mass spectrometer (Thermo Fisher Scientific, Bremen, Germany) equipped with a robotic nanoflow ion source TriVersa (Advion BioSciences Ltd, Ithaca NY) using chips with 4.1 µm nozzle diameter. The ion source was controlled by Chipsoft 6.4. software (Advion BioSciences) and operated at the ionization voltage of 0.95 kV and gas pressure 1.25 psi. Plates with lipid extracts were chilled down to 12°C.

MS survey scans were acquired in positive ion mode using the Orbitrap analyzer operated under the target mass resolution of 100,000 (Full Width at Half Maximum, FWHM), defined at *m/z* 400 under automatic gain control set to 1.0×10^6^ as the target value [24]. Where specified, targeted MS^n^ experiments were performed using collision-induced dissociation (CID) mode using the linear ion trap analyzer of the LTQ Orbitrap machine [24], [29].

### Data pre-processing and identification of lipids species

Raw data files acquired from analyzed samples were converted into *.mzXML format by readw.exe utility (a tool of Trans-Proteomic Pipeline software collection, downloaded from http://tools.proteomecenter.org/wiki/index.php?title=Software:ReAdW). Mass spectra were further processed by LipidX software developed in-house. Altogether, the data set consisted of 151 high-resolution survey mass spectra including 12 blank controls. Spectra acquired within 28 s to 120 s from the start of sample infusion (timing required to stabilize the analyte flow and electrospray, as was judged by total ion current (TIC) trace) were averaged and recalibrated using *m/z* of synthetic standards SM 35∶1 and PC –O 20∶0/-O 20∶0 as references. Recalibrated spectra were further aligned such that related peaks were matched within the full dataset. Only peaks detected at the signal-to-noise ratio above the factor of 5 and recognized in more than 20% of all spectra were further considered. Identification of lipid species relied on accurately determined masses [24] considering a mass accuracy of better than 4 ppm and a retrieval rate of 90% for all plasma samples. Peaks also recognized in blank controls were excluded. Lipidomics analysis covered 10 major lipid classes: cholesterol (Chol), cholesterylesters (Chol-FA), phosphatidylcholines (PC, PC-O), lysophosphatidylcholines (LPC), phosphatidylethanolamines (PE, PE-O), sphingomyelins (SM), diacylglycerols (DAG) and triacylglycerols (TAG).

### Lipid Quantification

Species of PC, PC-O and LPC were quantified by the intensity ratios of their peaks to the internal standard PC-O 18∶0/-O 18∶0; PE and PE-O species – to the internal standard PE-O 20∶0/-O 20∶0; SM species – to the internal standard SM 35∶1. The abundance of individual TAG, DAG and Chol-FA species was determined by dividing their absolute intensities with the average of the absolute intensities of the three internal standards.

The abundance of free cholesterol was determined from the intensity of the positively charged ammonium adduct at *m/z* 404.3892. As an integrated abundance measure of cholesteryl esters, we considered the intensity of the peak with *m/z* 369.3521 (Chol- moieties). This fragment is produced in the ion source from ammonium adducts of cholesteryl esters via neutral loss of acyl chains [18].

### Statistical analysis

Two-tailed bivariate Pearson correlation tests were applied to evaluate the correlation between plasma levels of total cholesterol and triglycerides as measured by routine clinical chemistry methods and sums of plasma levels of cholesterol and triglyceride species obtained by mass spectrometry.

To obtain a multivariate preliminary data survey a “Principal Component Analysis” of all 95 lipid species and Chol-moieties integral index was performed in order to assemble highly correlated lipid species into common factors. Using “Eigenvalues” over 3 and “Varimax” rotation with Kaiser Normalization five factors were extracted. Factor composition is shown in Supplemental Table S3. The association between lipidomic profile and hypertension was subsequently examined using a model of multivariate analysis of variance (MANOVA) with these factors as dependent variables and hypertension status as an independent factor, thereby regarding BMI and HOMA as covariates. The specific effect of each factor on hypertension status was then tested by univariate analysis of variance (ANCOVA) with *p*-values corrected for multiple testing (Bonferroni procedure).

In order to assess the impact of BMI and HOMA-IR on the plasma lipid profile of the study population, cut-offs for maximal discrimination of two groups were calculated by repeated discriminant function analysis using triglyceride species as discriminating variables. The resulting cutoffs were 27.5 kg/m^2^ for BMI (60^th^ percentile; 87.1% correct classification), and 3.5 for HOMA-IR (67^th^ percentile; 80.0% correct classification).

For comparisons among groups (insulin-sensitive vs. insulin-resistant and normotensive vs. hypertensive) a general linear model was applied with lipid species as dependent variables and HOMA or the hypertension status as fixed factor. Since most of the lipid species were correlated (by bivariate correlation analysis) to BMI, this parameter was used as covariate in both models evaluating the effects of insulin resistance and hypertension on lipid species. Additionally, HOMA was used as a covariate in the model calculating the effect of hypertension on lipid profile. Adjusted means of lipid species were taken from both models for further calculations.

Data are given as mean percentage changes or as mean with a 95% interval of confidence unless otherwise stated. A value of p<0.05 was considered statistically significant. All statistical analyses were performed with the SPSS statistical package (v.16.0 for Windows; SPSS, Chicago, IL, USA).

## Results

### Clinical characteristics of the study population

**Figure 1:**
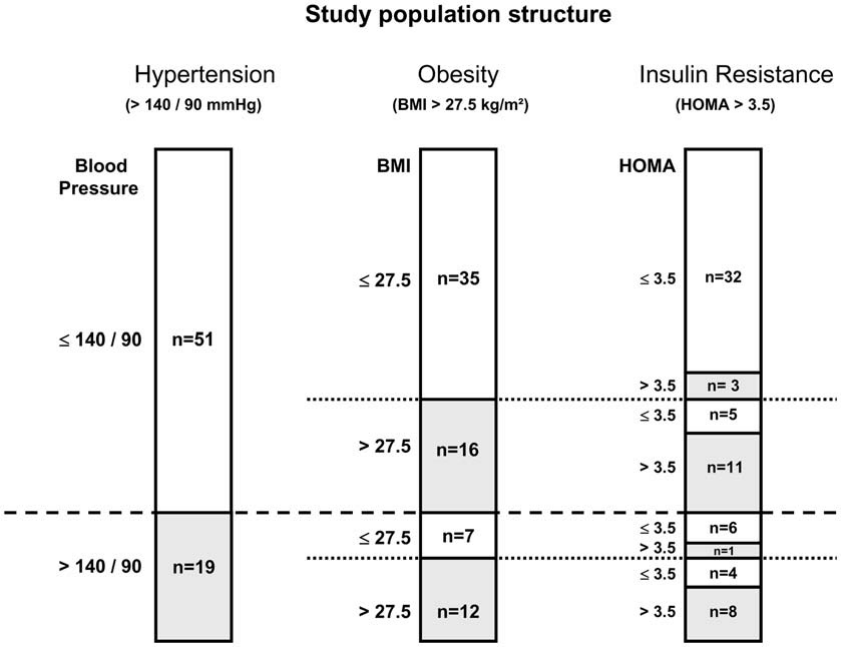
The study population. The structure of patient cohort is presented with respect to hypertension, obesity and insulin resistance. Bars represent the total number of patients (n = 70); horizontal dotted lines stand for the thresholds for blood pressure, BMI and HOMA-IR, respectively. Numbers within each bar indicate the number of patients within each of the sub-groups having the corresponding indices above or below the thresholds. For example, among hypertensive patients (n = 19; the bar at the left hand side), 7 were not obese (BMI<27.5 kg/m^2^; the bar in the middle) and 6 of those showed normal insulin resistance (HOMA-IR <3.5; the bar at the right hand side).

Basal clinical data show that men with hypertension had significantly higher BMI, WHR, fasting plasma glucose and HOMA (Table 1). The prevalence of hypertension was 16.7% (7/42) in lean and 42.9% (12/28) in obese men. Conversely, the prevalence of obesity (BMI>27.5 kg/m^2^) was 63.2% (12/19) in the hypertensive and 31.4% (16/51) in the control group (Figure 1). Furthermore, 47.4% (9/19) of the hypertensive group had a HOMA-IR index >3.5, whereby 8 were obese (Figure 1). In comparison, the prevalence of insulin resistance in the control group was 27.5% (14/51), including 11 obese individuals (Figure 1). Cumulatively, these data confirm an increased prevalence of obesity and insulin resistance in patients with hypertension, which were associated with significantly higher plasma triglyceride levels (Table 1). These differences disappeared after controlling the triglyceride values for BMI and HOMA.

**Table 1:** Basal anthropometric and clinical data in normotensive and hypertensive men.

		Normotensives n = 51	Hypertensives n = 19	Significance p =
**Age**	mean	51.7	57.3	0.173
[years]	95% CI	47.3–56.1	50.8–63.9	
**BMI**	mean	25.6	27.8	**0.012**
[kg/m^2^]	95% CI	24.7–26.5	26.3–29.3	
**WHR**	mean	0.92	0.96	**0.036**
	95% CI	0.90–0.94	0.92–0.99	
**RR systolic**	mean	125	154	**<0.001**
[mmHg]	95% CI	122–128	149–160	
**RR diastolic**	mean	72	85	**<0.001**
[mmHg]	95% CI	70–75	77–93	
**Triglycerides**	mean	1.28	1.65	**0.036**
[mM]	95% CI	1.13–1.43	1.24–2.06	
**Total** **cholesterol**	mean	5.11	5.20	0.691
[mM]	95% CI	4.89–5.34	4.79–5.62	
**HDL-cholesterol**	mean	1.53	1.48	0.614
[mM]	95% CI	1.42–1.63	1.32–1.63	
**LDL-cholesterol**	mean	3.31	3.30	0.945
[mM]	95% CI	3.09–3.53	2.81–3.78	
**Free fatty acids**	mean	0.46	0.54	0.105
[mM]	95% CI	0.41–0.52	0.47–0.61	
**HbA_1C_**	mean	5.4	5.6	0.299
[%]	95% CI	5.2–5.6	5.4–5.8	
**Glucose**	mean	5.3	5.6	**0.037**
[mM]	95% CI	5.1–5.5	5.3–6.0	
**Insulin**	mean	82	107	0.054
[pM]	95% CI	71–93	76–138	
**HOMA**	mean	2.87	3.91	**0.035**
	95% CI	2.43–3.30	2.77–5.05	

Statistical analyses by univariate analyses of variance.

### Shotgun screening of the plasma lipidome

**Figure 2:**
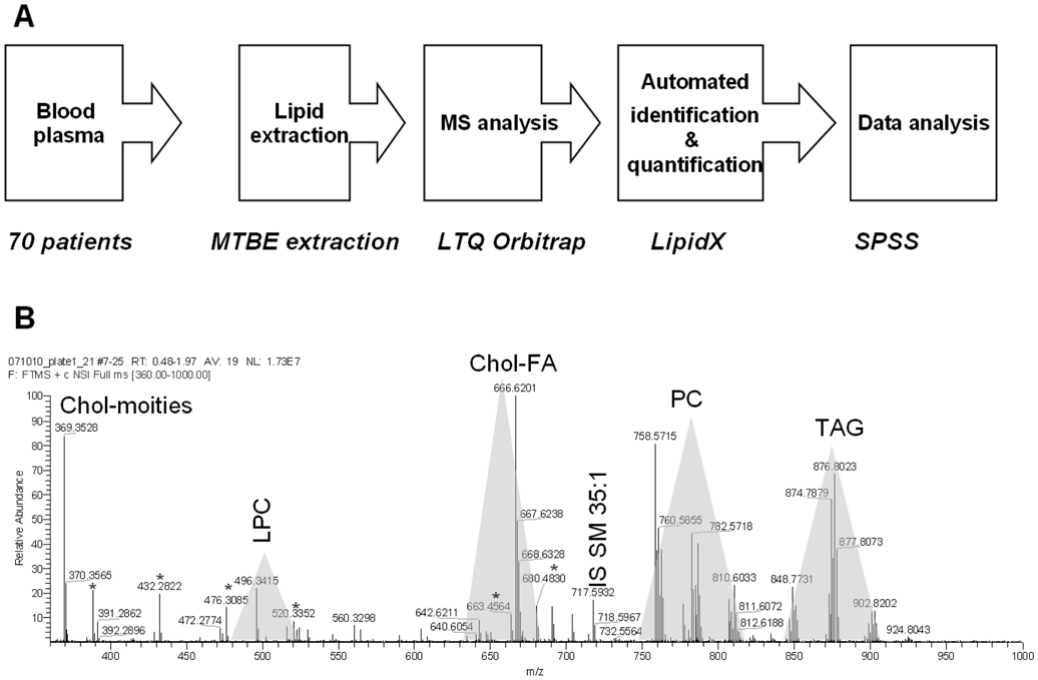
The workflow for top-down shogun lipidomic screens. A) EDTA plasma samples of 70 men (age of 22–79) was collected and lipids were extracted by methyl-*tert*-bytyl ether. Total extracts reconstituted in CHCl_3_/MeOH/2-propanol 1/2/4 (v/v/v) mixture, containing 7.5 mM ammonium acetate were directly infused into a LTQ Orbitrap mass spectrometer and high resolution mass spectra were acquired. 151 mass spectra were analyzed using LipidX software, which identified and quantified individual lipids. B) Representative high resolution mass spectrum of a total lipid extract of blood plasma. Spectra acquisition time was 3 min, while full sample analysis time was less than 4 min. Most abundant peaks are annotated with *m/z*; shaded areas designate *m/z* ranges in which corresponding lipid classes were detected. Major background peaks are designated with asterisks.

**Figure 3:**
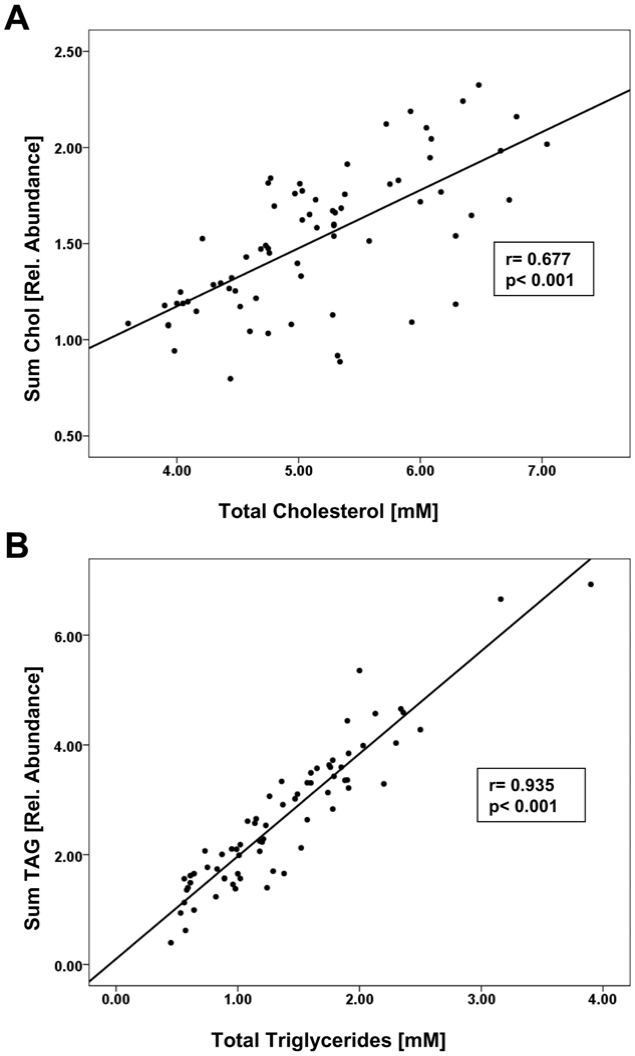
Quantification by top-down lipidomics correlates with clinical indices. A) Linear regression analysis of the total cholesterol content determined by mass spectrometry and by clinical chemistry analysis. Mass spectrometry quantified total cholesterol content by summing up the abundances of free cholesterol, all cholesteryl esters and their common fragment ion at *m/z* 369.35. B) Linear regression of the total triglyceride content determined by mass spectrometry and by clinical chemistry analysis. Mass spectrometry quantified the total TAG content by summing up all the abundances of individual TAG species. In both panels each dot represents the total extract of individual plasma sample.

A top-down shotgun lipidomics workflow [24] was optimized for high-throughput clinical screens (Figure 2). Internal standards were spiked into plasma samples prior to one-step lipid extraction by MTBE. Total lipid extracts were directly infused into a LTQ Orbitrap mass spectrometer and survey mass spectra acquired within less than 3 min time at the target mass resolution of 100,000 (FWHM). High mass resolution, better than 4 ppm mass accuracy and practical compositional constraints identified lipid species directly, solely relying on their accurately determined m/z [19], [24]. Each plasma sample was analyzed with 2 technical (independent analysis of the same extract) replicas. The dataset, organized in a form of a flat-file database by LipidX software, comprised 151 survey mass spectra acquired from 70 individual plasma samples. Within the imposed compositional constraints, LipidX quantified 95 lipids from 10 major classes: 5 lipids belonging to Chol-FA, 4 to DAGs, 5 to LPCs, 13 to PCs and 13 to PC-Os, 8 to PEs and 5 to PE-Os, 10 to SMs, 31 to TAGs, and free cholesterol. The total quantities of TAGs and cholesterol determined by mass spectrometry were strongly correlated with clinical indices (Figure 3).

### Principal component analysis (PCA) of the plasma lipidome

Multiple correlations of individual lipid species were analyzed by the method of PCA. Highly correlated lipid species were assembled into five common factors with Eigenvalues over 3 (Supplemental Table S3). Factor 1 included PCs, LPCs, and TAGs with short saturated fatty acid moieties; Factor 2 included all PC-O and PE-O species except PE-O [40∶5]; Factor 3 mostly included TAGs with polyunsaturated fatty acid moieties; Factor 4 mainly included SMs and cholesterol esters; Factor 5 mainly included TAGs and DAGs with unsaturated fatty acid moieties (Supplemental Table S3). The individual regression coefficients of these factors were saved and used as dependent variables in a model of multivariate analysis of variance (MANOVA).

### Both obesity and insulin resistance affect plasma lipidome

**Figure 4:**
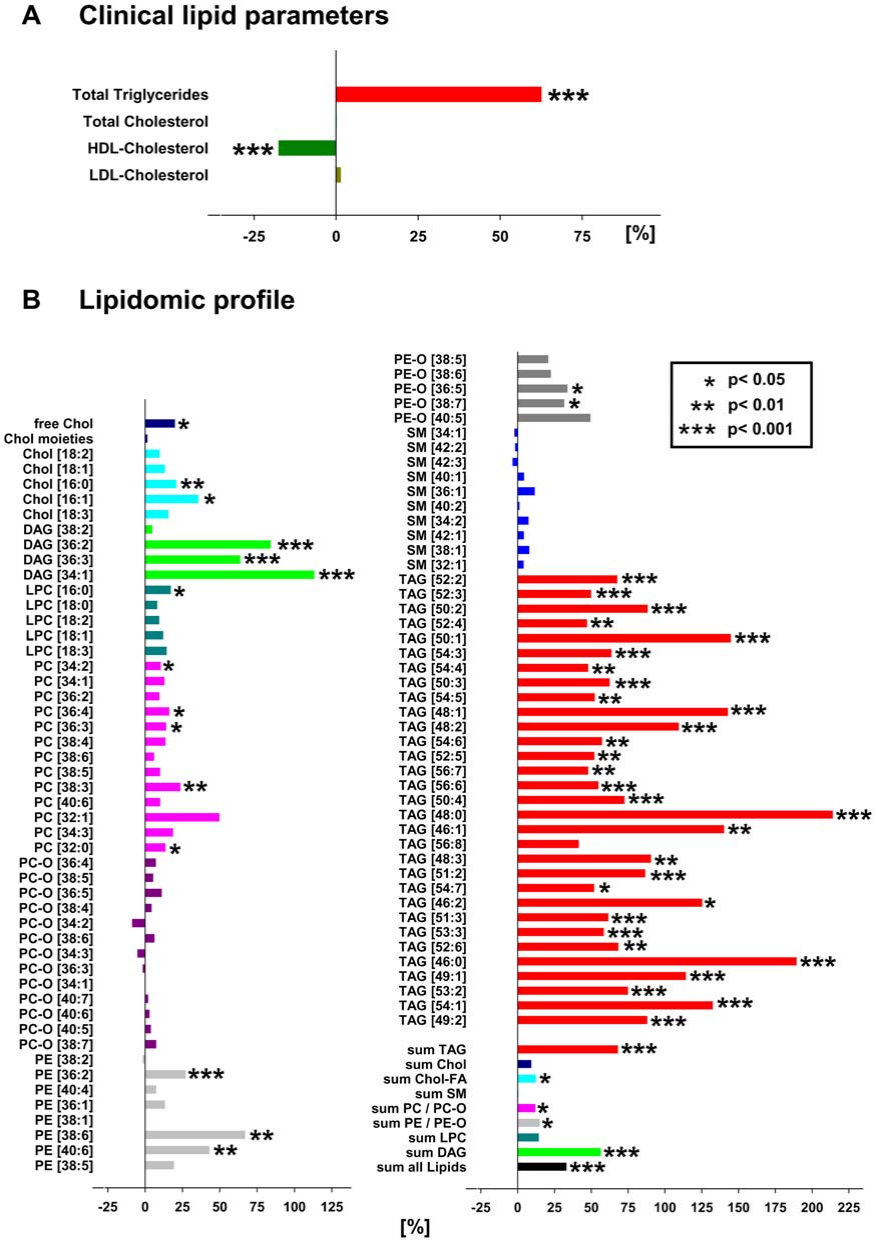
The effect of BMI increase on plasma lipidome. Changes in plasma lipidome of men with BMI >27.5 kg/m^2^ (n = 28) relative to a control group of men with BMI≤27.5 kg/m^2^ (n = 42), as determined by clinical indices (panel A) and by top-down shotgun mass spectrometry (panel B). In B relative % was determined for each species individually, irrespective of its absolute abundance. In the diagram, within each lipid class, species were sorted according to their absolute abundance from top to bottom in descending order. For example, among the TAG class the species TAG [52∶2] was the most abundant, while TAG [49∶2] was the least abundant. Data are presented as mean. Statistical analysis was performed by univariate analysis of variance.

Based on clinical chemistry data obese individuals had significantly higher triglycerides and lower HDL-cholesterol, whereas LDL- and total cholesterol remained unchanged (Figure 4A). Corroborating these findings, lipidomics analysis revealed that the abundance of nearly all lipids classes was generally increased (Figure 4B) in obese subjects. The content of saturated TAG and DAG species (Figure 4B) having, on average, less than one double bond per fatty acid moiety, increased 2- to 3- fold.

**Figure 5:**
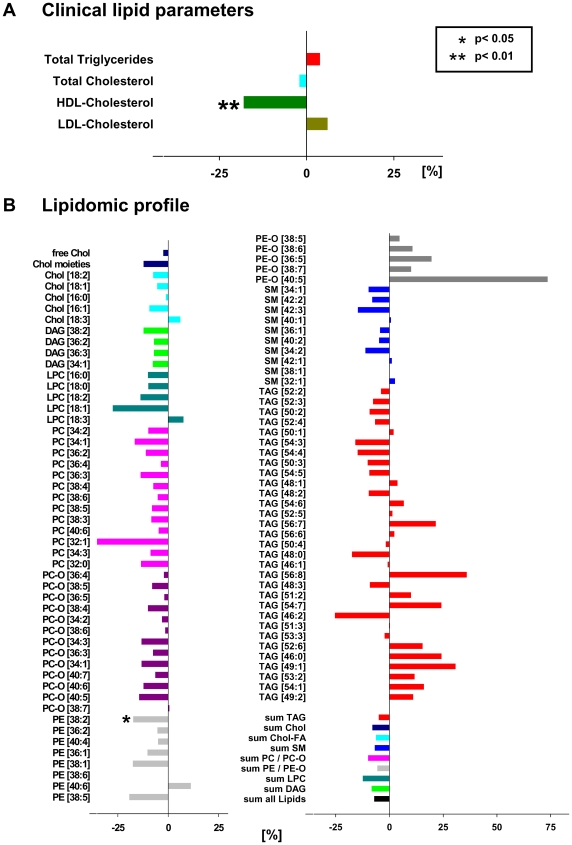
The effect of insulin resistance on plasma lipidome. Changes in plasma lipidome of men with HOMA-IR >3.5 (n = 23), relative to a control group of men with HOMA-IR≤3.5 (n = 47), as determined by clinical chemistry indices (panel A) and by top-down shotgun mass spectrometry (panel B). Statistical analysis by univariate analyses of variance with mean data controlled for BMI (ANCOVA) as described in Materials and Methods section.

According to clinical indices, individuals with insulin resistance (HOMA-IR >3.5) had a significant decrease in HDL-cholesterol, while changes in triglycerides and total cholesterol were insignificant (Figure 5A). The lipidomic screen (Figure 5B), controlled for the effects of BMI, revealed a pattern of predominantly decreased levels of LPC, PC, PC-O, PE, and SM, reaching significance only for PE 38∶2 species. There was also a clear trend toward increasing the content of PE-O species, while the content of TAGs varied considerably (Figure 5B). We therefore concluded that moderate insulin resistance is accompanied by specific, but minor changes of the lipidome, which might be partially concealed by the effects if obesity.

Altogether, shotgun lipidomics revealed that in the investigated cohort obesity and insulin resistance affect the lipidome in different ways. Since obesity-related changes are massive, special statistical considerations should be taken to reveal the effects of concomitant metabolic disorders.

### Hypertension and plasma lipidome

**Figure 6:**
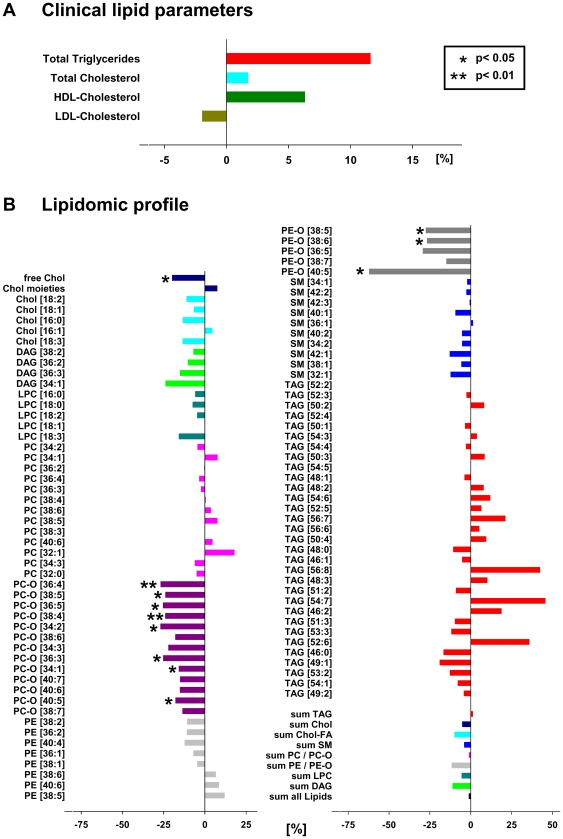
The effect of hypertension on plasma lipidome. Changes in plasma lipidome of men with hypertension (n = 19), relative to a control group of men without hypertension (n = 51), as determined by clinical indices (panel A) and by top-down shotgun mass spectrometry (panel B). Statistical analyses by univariate analyses of variance with mean data controlled for BMI and HOMA-IR (ANCOVA) as described in Material- and Methods section.

**Figure 7:**
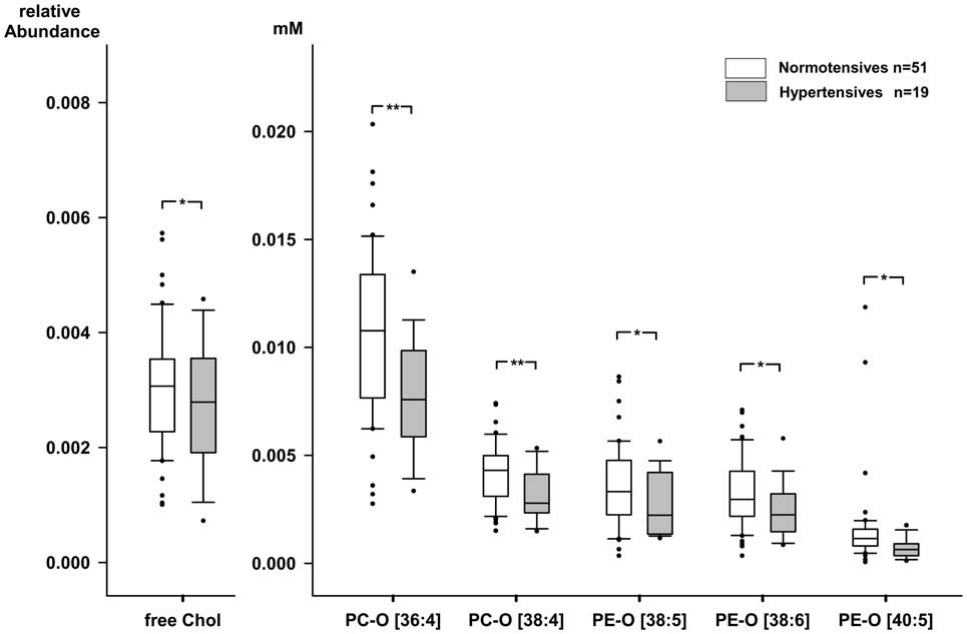
The effect of hypertension on selected species in the blood plasma lipidome. Box plots diagrams of plasma concentrations of free cholesterol, PC-O [36∶4], PC-O [38∶4], PE-O [38∶5], PE-O [38∶6], and PE-O [40∶5] in a group of men with hypertension (n = 19) and a control group of men without hypertension (n = 51) determined by top-down shotgun mass spectrometry. Statistical analyses by univariate analyses of variance. * p≤0.05, ** p≤0.01.

Contrary to the effect of BMI both clinical indices and shotgun profiling did not reveal major differences in plasma lipidome of normotensive and hypertensive subjects (Figure 6A and B), while changes of moderate magnitude that were observed among almost all lipid classes. The most notable exception, however, was a statistically confident and coherent decrease in the content of free cholesterol and PC-O species (Figures 6 and 7). Interestingly, the PE-O content was also decreased (significantly for the three out of five detected species, Figures 6 and 7), while PE and PC remained almost unchanged – which is, again, in sharp contrast to lipidomics changes instigated by BMI increase. Therefore, we concluded that hypertension is specifically associated with the deficiency of free cholesterol, PC-O, and PE-O species in male plasma lipidomes and it is seemingly unrelated to obesity-dependent alterations.

A multivariate model (MANOVA) comprising PCA factors as dependent variables, hypertension status as independent factor, and BMI and HOMA as covariates indicated significant and independent effects of BMI (p< 0.001) and hypertension status (p = 0.032). The subsequent univariate analysis with p-values corrected for multiple tests (Bonferroni procedure) appointed to the significant difference between normotensive and hypertensive individuals for Factor 2 (p = 0.018), which emphasized the role of ether lipids in the pathogenesis of hypertension.

Further, structural analysis of these PC-Os using with MS^3^ in the negative ion mode [30] revealed that all affected highly unsaturated species, such as PC-O 36∶4, PC-O 38∶5, PC-O 36∶5, PC-O 38∶4 and PC-O 38∶6 comprised the arachidonic acid moiety (Supplemental Table S2). We further determined that PC-O species were present in the concentration of approx. 32.2 µM (individual concentrations ranging from 9.6 to 64.8 µM) in the whole studied population. Individuals with hypertension had significantly lower levels of these PC-O species than normotensive controls (26.2±2.8 vs. 34.4±1.7 µM, p = 0.014). Structural analysis of downregulated PE-O species by MS^2^ in positive ion mode [31] revealed that these species were plasmalogens with high content of arachidonic acid moieties (Supplemental Table S2).

## Discussion

Hypertension, as an integral constituent of the metabolic syndrome, is frequently accompanied by obesity and insulin resistance, while both are known to strongly affect lipid metabolism [4], [5], [25], [26]. The analysis of the plasma lipidome by top-down shotgun mass spectrometry revealed that obesity increased the content of in Chol-FA, PC, PE, PE-O, and LPC species. Considering these changes in a model of univariate analysis of variance, we showed that hypertension was specifically associated with diminished free cholesterol and ether lipids levels that were statistically significant for eight PC-O and three PE-O species, in which arachidonic acid was the most abundant fatty acid moiety. It is therefore conceivable that the deficiency of arachidonic acid-rich ether lipids may substantially contribute to the pathogenesis of essential hypertension.

It is known that lipid concentrations in circulating blood show considerable gender-related differences. In premenopausal women total cholesterol, LDL-cholesterol and triacylglycerol concentrations are lower and HDL-cholesterol concentrations are higher than in men [32]. With age and menopausal transition, a profound shift in lipid profile occurs, which is characterized by an increase of LDL-cholesterol and triglycerides and a decrease in HDL-cholesterol. Sex hormone-induced effects on lipid profile may superpose and conceal pathogenetically relevant changes in lipidomic profiles. Therefore the subject selection of this study at this stage was restricted to men.

The association between BMI, insulin resistance, and hypertension was previously established [33]. Individuals with insulin resistance have a higher risk of developing hypertension [3]. Lipidomic profiles in the affected individuals are altered by BMI and insulin resistance [25]. In this study, individuals with hypertension had significantly higher values for BMI and HOMA-IR. Therefore, the effects of BMI and HOMA-IR on lipidomic profile were analyzed for the entire study population. Corroborating the evidence provided by Pietilainen et al [25], we observed that in individuals with a BMI >27.5 kg/m^2^ the content of almost all detectable TAG and DAG species increased, along with several Chol-FA, PCs, PEs, and LPCs. Additionally, the content of two PE-O species was significantly increased. Conversely, the group of individuals with insulin resistance (HOMA-IR >3.5) revealed a specific decrease in the abundance of PE 38∶2 species and the general tendency to diminished content of LPC, PC, PC-O, PE, SM, and PE-O species.

These alterations in lipid metabolism induced by increased fat cell mass and/or insulin resistance are likely to contribute to the development of the cardiovascular complications of the metabolic syndrome [3]. Elevations in inflammatory lipids such as LPC and long-chain fatty acids will induce endothelial dysfunction and eventually lead to hypertension [34]. Alterations in insulin secretion and other hormones such as aldosterone and catecholamines induced by these lipid changes will likewise affect blood pressure regulation [35]–[37].

A particular challenge in this study was to differentiate the r interrelationship of hypertension and lipid metabolism from concomitant effects of obesity and insulin resistance. Therefore, all quantities for lipid species were controlled for BMI and HOMA-IR using them as covariates in a general linear univariate model of variance (ANCOVA). This approach revealed a significant decrease of most of the analysed PC-O and PE-O lipids in individuals with hypertension, which was subsequently corroborated by direct absolute quantification of corresponding PC-O species.

Although a role for PC as major structural lipids of the cell membrane is well established, it is as yet unclear whether PC-O play any direct role in blood pressure control as has been previously suggested for structurally related platelet-activating factors (PAFs) [38], [39].

We emphasise that PC-Os identified in this screen are not PAFs as they harbour a fatty acid moiety at the *sn-*2 position instead of the acetyl moiety (Supplemental Table S2). The possibility remains that decreased abundances of PC-O and PE-O species reflect some enzymatic deficiency within the PAF biosynthesis pathway. In particular, lysophosphatidylcholine acyltransferase 2 (LPCAT2) is active in biosynthesis of both PC-Os and PAFs [40] and it is possible that observed changes in PC-Os are more pronounced because of their high stability and abundance in blood plasma, compared to PAFs.

Further structural characterization of PC-O and PE-O species revealed that the major molecular species were plasmalogens. Although Maeba et al. [41] reported reduced serum levels of plasmalogens in patients with abnormal glucose tolerance and coronary stenosis, their role in blood pressure regulation remains unknown.

Importantly, the affected PC-Os (36∶4, 38∶5, 36∶5, 38∶4) and PE-Os (38∶5, 38∶6, 40∶5) species are highly unsaturated and comprise arachidonic acid as a major fatty acid moiety. It is, however, unclear if their reduced content impacts on the activity of arachidonic acid metabolites, like prostaglandins (I_2_, E_2_) and epoxyeicosatrienoic acids (EET's), which play critical roles in the regulation of vascular tone [42]–[44], or reflects a masked deficit of arachidonic acid.

Free cholesterol residing in atherosclerotic plaques is an important factor leading to lesion instability [45], while its role in plasma remains unclear. Free plasma cholesterol was found in different lipoprotein particles (HDL, LDL, VLDL) [46]. However, the decrease of free plasma cholesterol in individuals with hypertension in our study was obviously not the result of changes in lipoproteins since the concentrations of HDL- and LDL-cholesterol did not differ between the groups. One possible explanation might be an increased activity of the lecithin:cholesterol acyltransferase (LCAT) in patients with hypertension. This suggestion is an accordance with the report from Dullaart et al. [47], which described increased LCAT activity in patients with metabolic syndrome.

Three important conclusions could be drawn from this study. First, top-down shotgun lipidomics established itself as a novel technology allowing high throughput clinical screens. Secondly, quantitative profiles of the blood plasma lipidome correlated well with clinical lipid homeostasis indices. Yet, they provide far more systematic and accurate description of metabolic disorders. Finally, this is the first study demonstrating a specific association between hypertension and lipid profiles. These results may form the basis for novel dietary strategies for the treatment of the metabolic syndrome and hypertension.

## Supporting Information

Table S1 Basal anthropometric and clinical data of the investigated population
(0.05 MB DOC)

Table S2 PC-O and PE-O lipid species showing significant decreased abundance in subjects with hypertension
(0.04 MB DOC)

Table S3 Principal component analysis of 95 lipid species and Chol-moieties integral index identified in blood plasma by mass spectrometry
(0.08 MB DOC)
