# Supplementary material for: Top-Down Lipidomics Reveals Ether Lipid Deficiency in Blood Plasma of Hypertensive Patients
Source: PLoS One. 2009 Jul 15;4(7):e6261. doi: 10.1371/journal.pone.0006261 (PMC2705678; doi:10.1371/journal.pone.0006261)
Supplement: Table S1 — Basal anthropometric and clinical data of the investigated population (0.05 MB DOC) [file pone.0006261.s001.doc]

**Supplemental Table A.** Basal anthropometric and clinical data of the investigated population (men, n= 70)

|  |  |
| --- | --- |
|  |  |
|  | **Mean ± SD** |
|  |  |
| Age [years] | 53.2 ± 15.2 |
|  |  |
| BMI [kg/m²] | 26.2 ± 3.3 |
|  |  |
| WHR | 0.93 ± 0.07 |
|  |  |
| RR systolic [mmHg] | 132.8 ± 17.1 |
|  |  |
| RR diastolic [mmHg] | 75.8 ± 12.2 |
|  |  |
| Triglycerides [mM] | 1.38 ± 0.65 |
|  |  |
| Total cholesterol [mM] | 5.14 ± 0.82 |
|  |  |
| HDL-cholesterol [mM] | 1.51 ± 0.36 |
|  |  |
| LDL-cholesterol [mM] | 3.31 ± 0.84 |
|  |  |
| Free fatty acids [mM] | 0.48 ± 0.18 |
|  |  |
| HbA_1C_ [%] | 5.5 ± 0.6 |
|  |  |
| Glucose [mM] | 5.4 ± 0.6 |
|  |  |
| Insulin [pM] | 89 ± 47 |
|  |  |
| HOMA | 3.15 ± 1.85 |
|  |  |
