# Supplementary material for: Top-Down Lipidomics Reveals Ether Lipid Deficiency in Blood Plasma of Hypertensive Patients
Source: PLoS One. 2009 Jul 15;4(7):e6261. doi: 10.1371/journal.pone.0006261 (PMC2705678; doi:10.1371/journal.pone.0006261)
Supplement: Table S2 — PC-O and PE-O lipid species showing significant decreased abundance in subjects with hypertension (0.04 MB DOC) [file pone.0006261.s002.doc]

**Supplemental Table B.** PC-O and PE-O lipid species showing significant decreased abundance in subjects with hypertension.

|  |  |  |  |  |  |  |
| --- | --- | --- | --- | --- | --- | --- |
| **Sum composition** | **Lipid species** | **(%)** | **Lipid species** | **(%)** | **Lipid species** | **(%)** |
| PC-O 36:4 | PC 16:0e / 20:4 | >95 |  |  |  |  |
| PC-O 36:5 | PC 16:0p / 20:4 | 90-95 | PC 16:0e / 20:5 | 5-10 |  |  |
| PC-O 38:4 | PC 18:0e / 20:4 | >95 |  |  |  |  |
| PC-O 38:5 | PC 18:0p / 20:4 | 75-90 | PC 16:0e / 22:5 | 10-20 | PC 18:0e / 20:5 | 5 |
| PC-O 34:2 | PC 16:0e / 18:2 | 65-75 | PC 16:0p / 18:1 | 25-35 |  |  |
|  |  |  |  |  |  |  |
|  |  |  |  |  |  |  |
| PE-O 38:5 | PE 18:0p / 20:4 | >85 | PE 16:0p / 22:4 | 5 - 10 | PE 18:1p / 20:3 | <5 |
| PE-O 38:6 | PE 18:1p / 20:4 | 45-70 | PE 16:0p / 22:5 | 10-30 | PE 18:0p / 20:5 | 5-25 |
| PE-O 40:5 | PE 20:0p / 20:4 | 45-80 | PE 18:0p / 22:4 | 15-55 |  |  |
|  |  |  |  |  |  |  |

Relative abundances of isobaric species of PC-O´s were determined from intensities of specific fatty acyl anions in MS^3^ in the negative ion mode. The relative abundances of isobaric PE-plasmalogens was determined from intensities of characteristic fragments of 1-*O*-alk-1’-enyl and 2-acyl moieties in MS^2^ in the positive ion mode.

Note: at the *sn*-1 position: *e*=1-*O*-alkylether, and *p*=1-*O*-alk-1′-enyl (plasmalogen)
