# Supplementary material for: Top-Down Lipidomics Reveals Ether Lipid Deficiency in Blood Plasma of Hypertensive Patients
Source: PLoS One. 2009 Jul 15;4(7):e6261. doi: 10.1371/journal.pone.0006261 (PMC2705678; doi:10.1371/journal.pone.0006261)
Supplement: Table S3 — Principal component analysis of 95 lipid species and Chol-moieties integral index identified in blood plasma by mass spectrometry (0.08 MB DOC) [file pone.0006261.s003.doc]

**Supplemental Table C.** Principal component analysis of 95 lipid species and Chol-moieties integral index identified in blood plasma by mass spectrometry

|  |  |  |  |  |  |
| --- | --- | --- | --- | --- | --- |
|  | **Factor 1** | **Factor 2** | **Factor 3** | **Factor 4** | **Factor 5** |
|  |  |  |  |  |  |
| **% of total variance** | **18.8 %** | **16.7%** | **15.1%** | **11.7%** | **11.0%** |
|  |  |  |  |  |  |
|  |  |  |  |  |  |
| **Order of factor loading** |  |  |  |  |  |
|  |  |  |  |  |  |
| 1 | PC [34:1] | PC-O [36:5] | TAG [56:7] | SM [34:1] | TAG [51:2] |
| 2 | PC [32:1] | PE-O [38:6] | TAG [54:6] | Chol [18:2] | TAG [53:2] |
| 3 | LPC [18:1] | PC-O [36:3] | TAG [54:7] | SM [40:1] | TAG [49:1] |
| 4 | Chol [16:1] | PC-O [34:3] | TAG [52:5] | SM [34:2] | TAG [49:2] |
| 5 | PC [38:5] | PC-O [38:5] | TAG [56:8] | SM [40:2] | TAG [51:3] |
| 6 | TAG [48:2] | PE-O [38:5] | TAG [54:5] | Chol [18:1] | DAG [36:2] |
| 7 | TAG [46:1] | PC-O [36:4] | TAG [52:4] | SM [38:1] | TAG [53:3] |
| 8 | TAG [46:2] | PC-O [40:6] | TAG [56:6] | SM [32:1] | TAG [52:2] |
| 9 | TAG [48:1] | PC-O [38:6] | TAG [52:6] | SM [42:1] | DAG [34:1] |
| 10 | LPC [16:0] | PC-O [40:5] | PE [40:6] | PC [34:2] | PE [36:2] |
| 11 | TAG [48:0] | PC-O [34:2] | TAG [50:4] | Chol moieties | PE-O [40:5] |
| 12 | PC [36:4] | PE-O [36:5] | TAG [54:4] | SM [42:2] |  |
| 13 | TAG [50:2] | PC-O [38:4] | PC [40:6] | Chol [16:0] |  |
| 14 | PC [32:0] | PC-O [38:7] | PC [38:6] | SM [36:1] |  |
| 15 | TAG [48:3] | PC-O [34:1] | TAG [52:3] | free Chol |  |
| 16 | PC [34:3] | PE-O [38:7] | DAG [36:3] | PC [36:2] |  |
| 17 | PC [38:4] | PC-O [40:7] | PE [38:6] | DAG [38:2] |  |
| 18 | TAG [46:0] | SM [42:3] |  | Chol [18:3] |  |
| 19 | PE [36:1] | PE [38:2] |  |  |  |
| 20 | TAG [50:1] | PE [40:4] |  |  |  |
| 21 | TAG [54:1] |  |  |  |  |
| 22 | PC [36:3] |  |  |  |  |
| 23 | PE [38:5] |  |  |  |  |
| 24 | TAG [50:3] |  |  |  |  |
| 25 | LPC [18:0] |  |  |  |  |
| 26 | LPC [18:3] |  |  |  |  |
| 27 | TAG [54:3] |  |  |  |  |
| 28 | PC [38:3] |  |  |  |  |
| 29 | PE [38:1] |  |  |  |  |
| 30 | LPC [18:2] |  |  |  |  |
|  |  |  |  |  |  |

Factors with Eigenvalues over 3 were extracted. Rotation method: Varimax with Kaiser normalization. Total variance explained: 73.3%. Metabolites in decreasing order of factor loadings (1 = highest)
